# Phase I study of KRP‐116D, a 50% w/w dimethyl sulfoxide aqueous solution, on the systemic absorption from bladder by intravesical instillation in healthy Japanese subjects

**DOI:** 10.1111/luts.12295

**Published:** 2019-11-06

**Authors:** Hideyo Shimada, Makoto Yono, Yuya Hojo, Yuka Hamamura, Atsushi Ootsuki

**Affiliations:** ^1^ Department of internal medicine Shimada Medical Clinic Tokyo Japan; ^2^ Department of Clinical Pharmacology Nishi Kumamoto Hospital, Souseikai Kumamoto Japan; ^3^ Department of DMPK Research Laboratory Kyorin Pharmaceutical Co., Ltd. Tochigi Japan; ^4^ Department of Clinical Development Kyorin Pharmaceutical Co., Ltd. Tokyo Japan

**Keywords:** dimethyl sulfoxide, interstitial cystitis/bladder pain syndrome, intravesical instillation, KRP‐116D, pharmacokinetic

## Abstract

**Objective:**

This was a single‐institution, single‐dose, single‐arm phase 1 study in healthy adult males to evaluate the safety and absorption of dimethyl sulfoxide (DMSO) from the bladder into the body when KRP‐116D (a 50% w/w DMSO solution) was intravesically administered and allowed to remain in the bladder for 15 minutes.

**Methods:**

Six healthy adult males were enrolled in this study. KRP‐116D (50 mL) was instilled directly into the bladder via a catheter where it was allowed to remain for 15 minutes under lidocaine anesthesia in accordance with the usage of RIMSO‐50 (50% w/w DMSO solution) approved in the USA. The residual DMSO solution in the bladder was collected 15 minutes after instillation. The concentrations of DMSO in the plasma and the recovered solution were analyzed by a validated high‐performance liquid chromatography (HPLC) method. The concentration in the residual DMSO solution was multiplied by the solution volume and divided by the dosage to calculate the recovery rate of DMSO.

**Results:**

Plasma DMSO was detected in one of six subjects, and in the remaining five subjects DMSO was not detected (<19.6 μg/mL). The recovery rate of DMSO from the bladder was 60.7% to 93.7%. The only drug‐related adverse event was breath odor (garlic‐like breath) observed in four of six subjects (66.7%).

**Conclusion:**

Absorption of DMSO from the bladder was low (16.3%), and the systemic exposure was limited. Most of the DMSO was recovered from the bladder. KRP‐116D 50 mL was well tolerated and safe.

## 1 INTRODUCTION

Interstitial cystitis (IC), also referred to as bladder pain syndrome (BPS), is a chronic inflammatory disease of the bladder associated with bladder hypersensitivity, urinary frequency, and bladder pain in the absence of other well‐defined pathologies such as urinary tract infection or malignancy.^1^, ^2^, ^3^ In Japan, the high prevalence (265 per 100 000) in female patients is similar to the prevalence in Western countries reported in a Web‐based survey^4^; however, a recent survey estimated that about 4500 IC patients and 2000 Hunner‐type IC patients require some form of medical care.^5^ IC/BPS has a significant negative impact on the quality of life in several psychosocial dimensions including vitality and mental health.^6^ However, the drugs approved by the US Food and Drug Administration for IC/BPS are confined to pentosan polysulfate sodium and a 50% w/w solution of dimethyl sulfoxide (50% DMSO). The treatment algorithm of the American Urological Association (AUA) Guideline for IC/BPS recommends the intravesical instillation of 50% DMSO as a second‐line treatment for patients with IC/BPS following first‐line treatment that includes general relaxation/stress management, pain management, self‐care/behavioral management, and patient education.^2^ Intravesical treatment with DMSO is also listed in the Japanese guideline on the diagnosis and treatment of IC,^1^ but has not yet been approved in Japan.

Although the mechanism of action in IC/BPS remains unclear, DMSO is known to have a variety of pharmacological activities such as membrane penetrant, a “carrier” of solutes across membranes; anti‐inflammatory, analgesic, and diuretic effects; cholinesterase inhibition, muscle relaxation, and vasodilation, resulting in improvement of the disease.^7^, ^8^ Kyorin Pharmaceutical Co., Ltd., (Tokyo, Japan) planned the development of 50% DMSO (code name: KRP‐116D) for the treatment of IC/BPS on the basis of a request from the Committee on Unapproved Drugs and Drugs of Off‐label Use Urgently Required for Healthcare organized by the Ministry of Health, Labour and Welfare. Accordingly, the company has been conducting a randomized placebo‐controlled phase 3 trial in Japan.

There are reports that a garlic‐like taste is noted by the patient within a few minutes after intravesical instillation of 50% DMSO, which persists for several hours, and that an odor on the breath and skin may remain for 72 hours.^9^ This suggests that DMSO is absorbed from the bladder into the body. However, to the best of our knowledge, there is no literature describing the absorption of intravesical instillation of 50% DMSO in humans. Here, we report on a phase 1 clinical trial of KRP‐116D in healthy adult males designed to evaluate the safety and assumed absorption of DMSO from the bladder into the body when KRP‐116D is intravesically administered and allowed to remain in the bladder for 15 minutes.

## 2 METHODS

### 2.1 Study design and subjects

This was a single‐institution, single‐dose, single‐arm phase 1 study. KRP‐116D is a 50% w/w DMSO sterile solution manufactured in accordance with good manufacturing practice (GMP) by Kyorin Pharmaceutical Co., Ltd. The study was undertaken at the Department of Clinical Pharmacology, Nishi Kumamoto Hospital, Kumamoto, Japan. The study protocol and informed consent form were approved by the Hakata Clinic Institutional Review Board (held on 13 April 2018; management number L‐57). All subjects gave written informed consent before the initiation of any study‐specific procedure. The study was conducted in accordance with the ethical principles originating in or derived from the Declaration of Helsinki, Good Clinical Practice Guidelines, and locally applicable laws and regulations.

Inclusion criteria were: healthy Japanese male subject, aged 20 to 35 years, a body mass index (BMI) of 18.5 to 25.0 kg/m^2^, and confirmation of health by an investigator based on medical examination, observation, and clinical laboratory tests. Subjects were required to be of Japanese ancestry going back at least two generations (grandparents). IC/BPS is much more common in female patients than male patients,^4^ but male adults were selected as subjects since the risk of drug leakage from the bladder is very limited due to the longer urethra than in women, and there is no risk of pregnancy. Only young male adults were enrolled in order to remove the factors affected by complications and aging from this study.

Major exclusion criteria were history of severe allergic reactions, atopy, anaphylaxis, or intolerance to drugs or foods; any history of disease or surgery that would preclude being enrolled in the study; hypersensitivities to DMSO or amide‐type local anesthetic agents; excessive alcohol consumption or not abstaining from alcohol during the specified study period; heavy smoking or not abstaining from smoking during the specified study period; excessive coffee intake or ingesting caffeine‐containing foods every day or not abstaining from such foods during the study period; having medical treatment 4 weeks prior to instillation, use of over‐the‐counter drugs 2 weeks prior to instillation, or possible use of prohibited medication during the study; whole blood collection of ≥800 mL within 1 year, ≥400 mL within 12 weeks, or 200 mL within 4 weeks, or blood component donation within 2 weeks before screening; hemoglobin <13.5 g/dL at screening or the first day of hospitalization; and being positive for hepatitis B (HB) antigen, hepatitis C virus (HCV) antibody, HIV antigen or antibody, or syphilis test at screening. The enrollment of six subjects was considered a sufficient number to achieve the study objectives.

### 2.2 Treatment and assessments

A total of six subjects who met the eligibility criteria were enrolled in the study. The day of study drug administration was designated as “day 1”, and after the screening period (day ‐28 to day ‐2), subjects entered the study unit on day ‐1 and were hospitalized until day 3. Before instillation of KRP‐116D, residual urine in the bladder was removed using a urethral catheter. Lidocaine solution (4%, 20 mL) was instilled directly into the bladder, allowed to remain for 15 minutes for bladder anesthesia, and the residual lidocaine solution was removed from the bladder using a urethral catheter. Subjects received a single dose of KRP‐116D (50 mL) intravesically through a urethral catheter that was then allowed to remain in the bladder for 15 minutes. Next, the residual DMSO solution was collected from the bladder using a urethral catheter, and the recovered volume (mL) was measured. Blood samples for the measurement of plasma DMSO were taken immediately pre dose, and then 0.25, 0.5, 1, 2, 4, 8, 12, 24 (day 2), 36 (day 2), and 48 (day 3) hours post dose. DMSO concentrations in the plasma and recovered DMSO solution were measured by a validated high performance liquid chromatography (HPLC) method. The below lower limit of quantification (BLQ) of DMSO in the plasma and recovered DMSO solution was 19.6 μg/mL and 391 μg/mL, respectively. Pharmacokinetic (PK) parameters for DMSO included maximum plasma concentration (C_max_), time to maximum plasma concentration (t_max_), elimination half‐life (t_1/2_), and area under the concentration‐time curve (AUC) from time zero to the last measurable concentration (AUC_last_), all of which were calculated by noncompartmental analysis using Phoenix WinNonlin Version 7.0 (Certara, Princeton, New Jersey).

Safety was assessed in relation to adverse events (AEs), clinical laboratory tests (hematology, chemistry, and urinalysis), 12‐lead electrocardiogram (ECG), vital signs (systolic and diastolic blood pressure, pulse rate, and temperature), and physical examinations. Hematology consisted of leukocyte count, leukocyte fraction (neutrophil, lymphocyte, monocyte, eosinophil, basophil), red blood cell count, hematocrit value, hemoglobin amount, and platelet count. Chemistry consisted of aspartate aminotransferase (AST), alanine aminotransferase (ALT), alkaline phosphatase (Al‐P), lactate dehydrogenase (LDH), γ‐glutamyl transpeptidase (γ‐GTP), total bilirubin (T‐Bil), direct bilirubin (D‐Bil), creatine kinase (CK), total protein (TP), triglyceride (TG), blood glucose, total cholesterol (T‐Cho), albumin (Alb), blood urea nitrogen (BUN), creatinine (Cr), uric acid (UA), and electrolytes including sodium (Na), potassium (K), chloride (Cl), calcium (Ca), and phosphorus (P). Urinalysis consisted of qualitative analysis including protein, glucose, and urobilinogen and sediments of red blood cells and white blood cells. Criteria for abnormal variation in clinical laboratory tests are shown in Table S1. Blood samples for clinical laboratory tests were taken at screening, day ‐1, and 0 (pre dose), 1, 24, and 48 hours post dose. The investigators asked the subjects how they felt using nonleading questions at the daily physical examination. The subjects were also asked to self‐report AEs at any time to any staff member including the investigator or nurses. The PK analysis set was defined as subjects who received the study drug and who gave a blood sample for PK analysis and whose residual solution was collected from the bladder. The safety analysis set was defined as subjects who received the study drug and had safety assessment data.

## 3 RESULTS

Six healthy adult male subjects were enrolled and all completed the study. The mean age was 23.8 ± 4.7 years (range: 20–32 years), the mean body weight was 68.43 ± 11.73 kg (range: 47.6–83.5 kg), and the mean BMI was 22.53 ± 2.08 kg/m^2^ (range: 18.8–24.9 kg/m^2^).

All subjects received a single dose (50 mL) of KRP‐116D, corresponding to approximately 27 g of DMSO as an active ingredient, which was allowed to remain in the bladder for 15 minutes, and all subjects were included in the PK analysis set and safety analysis set. There were no major protocol deviations.

### 3.1 Assumed DMSO absorption from the bladder

The amount of recovered DMSO from the bladder and the recovery rate of each subject are shown in Table 1. The mean amount of recovered DMSO and percentage were 22.52 ± 3.22 g (median: 23.46, range: 16.3–25.2 g) and 83.7 ± 12.0% (median: 87.2, range: 60.7‐93.7%), respectively. The mean assumed amount of absorbed DMSO and percentage from the bladder to the body were 4.38 ± 3.22 g (median: 3.44, range: 1.71–10.6 g) and 16.3 ± 12.0% (median: 12.8, range: 6.34‐39.3%), respectively.

**Table 1:** The recovered DMSO (g) and its recovery rate (%) in the recovered solution of the study drug from the bladder and the pharmacokinetic parameters of each patient who received a single dose (50 mL) of KRP‐116D

Subject no	Recovered solution		PK parameters (plasma)		
	DMSO(g)	Recovery rate (%)	C_max_ a(μg/mL)	t_1/2_(h)	AUC_last_(μg･.h/mL)
1	16.3	60.7	72.1	5.46	150
2	22.2	82.5	ND	ND	ND
3	24.5	91.1	ND	ND	ND
4	25.2	93.7	ND	ND	ND
5	22.9	85.2	ND	ND	ND
6	24.0	89.2	ND	ND	ND

Abbreviations: AUC_last_, area under the concentration‐time curve (AUC) from time zero to the last measurable concentration; C_max_, maximum plasma concentration; DMSO, dimethyl sulfoxide; ND, not detected; PK, pharmacokinetic; t_1/2_, elimination half‐life.

a The below lower limit of quantification (BLQ) of DMSO in the plasma and recovered solution were 19.6 μg/mL and 391 μg/mL, respectively.

### 3.2 Pharmacokinetics

Among six subjects, the plasma DMSO concentrations of five subjects were not detected (under BLQ). The C_max_, t_max_, t_1/2_, and AUC_last_ of one subject were 72.1 μg/mL, 0.250 hours, 5.46 hours, and 150 μg･.h/mL, respectively. Summary statistics were not available because plasma PK parameters were obtained in only one subject.

### 3.3 Safety

Among six subjects, four AEs occurred in four subjects (66.7%). All these events were breath odor (Table 2). These events were objective not subjective findings. In all cases the severity was mild. The investigator determined that a causal relationship with the study drug could not be ruled out. No AEs due to the intravesical instillation procedure itself were observed. There were no deaths, serious AEs, or AEs leading to discontinuation. No notable changes in clinical laboratory tests, vital signs, and 12‐lead ECG were observed during the study.

**Table 2:** Summary of adverse events (safety analysis set; N = 6)

	Event, n	Subject, n (%)
Adverse event	4	4 (66.7)
Breath odor	4	4 (66.7)
Drug‐related adverse event	4	4 (66.7)
Breath odor	4	4 (66.7)
Serious adverse event	0	0
Drug‐related serious adverse event	0	0
Adverse event leading to discontinuation	0	0
Drug‐related adverse event leading to discontinuation	0	0

## 4 DISCUSSION

Intravesical instillation of 50% w/w DMSO solution has been a mainstay of pharmacological treatment for IC/BPS in the USA and Canada for nearly 40 years since its approval in 1978 in the USA. Currently, 50% w/w DMSO solution is recommended for use as a second‐line treatment in the AUA Guideline for IC/BPS. Dosage and treatment of DMSO in the US package insert of RIMSO‐50 (Mylan Pharmaceuticals Inc., Pennsylvania, USA) states “instillation of 50 mL of RIMSO‐50® (dimethyl sulfoxide) directly into the bladder may be accomplished by catheter or asepto syringe and be allowed to remain in situ for 15 minutes. Application of an analgesic lubricant gel such as lidocaine jelly to the urethra is recommended prior to insertion of the catheter to avoid spasm. The medication is expelled by spontaneous voiding.”^9^ The extensive experience in patients with IC/BPS in the USA suggests that 50 mL of 50% DMSO would also be safe in Japanese patients. On the other hand, no studies have examined absorption of DMSO from the bladder into the body. Therefore, we planned to evaluate the absorption and safety of KRP‐116D in Japanese subjects using the same dose and treatment method as recommended in the US package insert of RIMSO‐50. According to the package insert, the instilled lidocaine preparation is expelled by spontaneous voiding, but in this study, it was removed using a catheter in order to standardize the treatment conditions among the subjects. DMSO was detected in plasma in one subject, and not detected in the remaining five subjects when KRP‐116D (50 mL) was directly instilled into the bladder and allowed to remain in the bladder for 15 minutes. The reason why DMSO was absorbed from the bladder in only one among six subjects was unknown. The DMSO recovery rate in the subject in whom DMSO was detected in plasma was lowest (60.7%) compared with those (range: 82.5‐93.7%) in whom DMSO was not detected. This finding suggests that DMSO was absorbed from the bladder, and the rate of absorption into the body may be related to the plasma concentration of DMSO. Hucker et al^10^ reported that orally administered DMSO in men was rapidly absorbed, reaching a peak in serum in 4 hours. The reported systemic exposure of DMSO orally administered was much higher than that of intravesically administered DMSO, suggesting absorption of DMSO from the bladder was much less than that of oral administration.^10^ Incidentally, the main metabolites, dimethyl sulfone and dimethyl sulfide, were not analyzed in our study.

Among six subjects, four AEs occurred in four subjects. All events were breath odor (garlic‐like breath) and were judged as a drug‐related AE. The breath odor might be due to a metabolite, dimethyl sulfide. It should be noted that the findings for this event were objective, not subjective; in other words, the smell itself was clearly detected by the investigator and clinical staff, but the subjects themselves were not aware of it. In this study, because the subjects were hospitalized during the study, this made it particularly likely that objective findings would to be detected because the study staff was strictly conducted over time in accordance with the study protocol. These findings suggest that the incidence of this AE may differ depending on the subject's environment. Breath odor was not reported in the subject in whom DMSO was detected in plasma. Interestingly, there was no relationships between plasma DMSO concentration and breath odor. Garlic‐like breath odor might be due to a metabolite, dimethyl sulfide. However, the main metabolites, dimethyl sulfone and dimethyl sulfide, were not analyzed in our study.

## 5 CONCLUSION

Plasma DMSO concentration was detected in only one of six subjects and was not detected in the remaining five subjects when the subjects received intravesical instillation with a single dose of 50% w/w DMSO solution. Most DMSO was recovered from the bladder. The absorption rate of DMSO from the bladder was low (16.3%), and the systemic exposure was limited. KRP‐116D 50 mL was well tolerated and safe.

## CONFLICT OF INTEREST

Kyorin Pharmaceutical Co., Ltd. received a grant from the National Institutes of Biomedical Innovation, Health and Nutrition. Hideyo Shimada MD received a consultancy fee from Kyorin Pharmaceutical Co., Ltd. Makoto Yono has nothing to declare. Yuya Hojo, Yuka Hamamura, and Atsushi Ootsuki are employees of Kyorin Pharmaceutical Co., Ltd.

## Supporting information


**Table S1.** Criteria for abnormal variation in clinical laboratory tests.
