# Supplementary material for: Phase I study of KRP‐116D, a 50% w/w dimethyl sulfoxide aqueous solution, on the systemic absorption from bladder by intravesical instillation in healthy Japanese subjects
Source: Low Urin Tract Symptoms. 2019 Nov 6;12(2):150–4. doi: 10.1111/luts.12295 (PMC7217045; doi:10.1111/luts.12295)
Supplement: Supplementary file 1 — Table S1. Criteria for abnormal variation in clinical laboratory tests. [file LUTS-12-150-s001.docx]

**TABLE S1.** Criteria for abnormal variation in clinical laboratory tests.

| Test item | | Abnormal variation | |
| --- | --- | --- | --- |
|  |  | Decrease | Increase |
| Hematology | Leukocyte count | ＜3000 /mm^3^ | ‒ |
|  | Neutrophil | ＜1500 /mm^3^ | ‒ |
|  | Lymphocyte | ＜800 /mm^3^ | ＞4000 /mm^3^ |
|  | Monocyte | Judgment by the investigator | |
|  | Eosinophil | ‒ | ≧500 /mm^3^ or ≧10% |
|  | Basophil | Judgment by the investigator | |
|  | Red blood cell count | ＜350 × 10^4^/mm^3^ | ‒ |
|  | Hematocrit value | ＜35% | ‒ |
|  | Hemoglobin amount | ＜10 g/dL | ‒ |
|  | Platelet count | ＜7.5 × 10^4^/mm^3^ | ≧60 × 10^4^/mm^3^＋symptom or ≧100 × 10^4^/mm^3^ |
| Chemistry | Aspartate aminotransferase (AST) | ‒ | ＞2.5 × ULN |
|  | Alanine aminotransferase (ALT) | ‒ | ＞2.5 × ULN |
|  | Alkaline Phosphatase (Al-P) | ‒ | ＞2.5 × ULN |
|  | Lactate dehydrogenase (LDH) | ‒ | ＞2.5 × ULN |
|  | γ-Glutamyl transpeptidase (γ-GTP) | ‒ | ＞2.5 × ULN |
|  | Total bilirubin (T-Bil) | ‒ | ≧1.5 × ULN |
|  | Direct bilirubin (D-Bil) | ‒ | ≧1.5 × ULN |
|  | Creatine kinase (CK) | ‒ | ＞2.5 × ULN |
|  | Triglyceride (TG) | ‒ | ＞300 mg/dL |
|  | Blood glucose | ＜55 mg/dL | ＞160 mg/dL |
|  | Total cholesterol (T-Cho) | ‒ | ＞300 mg/dL |
|  | Total protein (TP) | Judgment by the investigator | |
|  | Albumin (Alb) | ＜3 g/dL | ‒ |
|  | Blood urea nitrogen (BUN) | ‒ | ≧1.5 × ULN |
|  | Creatinine (Cr) | ‒ | ≧1.5 × ULN |
|  | Uric acid (UA) | Judgment by the investigator | |
|  | Sodium (Na) | ≦125 mEq/L | ≧155 mEq/L |
|  | Potassium (K) | ≦3.2 mEq/L | ≧5.5 mEq/L |
|  | Chloride (Cl) | ≦96 mEq/L | ≧115 mEq/L |
|  | Calcium (Ca) | ＜8.0 mg/dL | ＞11.5 mg/dL |
|  | Phosphorus (P) | ＜2.5 mg/dL | ‒ |
| Urinalysis | Protein qualitative | Two or more stages of fluctuation including ± as a stage | |
|  | Sugar qualitative | Two or more stages of fluctuation including ± as a stage | |
|  | Urobilinogen qualitative | Judgment by the investigator | |
|  | Red blood cell sediment | Judgment by the investigator | |
|  | Leukocyte sediment | Judgment by the investigator | |

ULN：Upper limit of normal
